# *Bifidobacterium longum* Subspecies *infantis* M63 Enhances the Colitis-Alleviating Effect of 2′-Fucosyllactose by Restoring Intestinal Barrier Function and Microbial Metabolism

**DOI:** 10.3390/foods15173113

**Published:** 2026-09-01

**Authors:** Hongwei Zhang, Xin Li, Muqiu Tan, Yihong Bao, Shilong Jiang

**Affiliations:** 1 Feihe Research Institute, Heilongjiang Feihe Dairy Co., Ltd., Beijing 100015, China; zhanghongwei@neau.edu.cn (H.Z.);; 2 College of Food and Health, Northeast Forestry University, Harbin 150040, China; 3 College of Food Science, Northeast Agricultural University, Harbin 150030, China

**Keywords:** synbiotics, colitis, cytokines, barrier function, gut microbiota, metabolism

## Abstract

Both 2′-fucosyllactose (2′-FL) and *Bifidobacterium* can modulate gut health; however, whether their combination provides strain-dependent protection against colitis remains unclear. This study aimed to compare the protective effects of 2′-FL alone and in combination with *Bifidobacterium longum* subsp. *infantis* M63 or *Bifidobacterium breve* M16V in dextran sulfate sodium (DSS)-induced colitis. Forty-eight male C57BL/6J mice were randomly assigned to six groups: negative control, DSS model control, 5-aminosalicylic acid positive control, 2′-FL, 2′-FL + M63, and 2′-FL + M16V. The interventions were administered for two weeks, with 2.5% DSS supplied in the drinking water during the second week. Colitis-related indices, colonic histopathology, inflammatory cytokines, oxidative-stress markers, intestinal barrier-associated gene expression, gut microbiota composition, lactate, and short-chain fatty acids (SCFAs) were evaluated. Compared with the model control, 2′-FL and both synbiotic combinations ameliorated colonic tissue injury and goblet-cell loss. Both combinations significantly reduced IL-1β, whereas 2′-FL and both combinations reduced IL-6 and TNF-α. The combinations also increased catalase and glutathione peroxidase activities and decreased malondialdehyde levels. 2′-FL alone increased *Occludin* and *RELMβ* expression, whereas 2′-FL + M63 showed the strongest effects on tight-junction and mucus-barrier restoration, including increased *Claudin-1*, *Claudin-2*, *ZO-1*, *TFF3*, and MUC2 expression. All 2′-FL-containing interventions increased microbial diversity, while 2′-FL + M63 increased *Bacteroidota*, reduced *Proteobacteria*, and enriched *Muribaculaceae* and *Ruminococcaceae.* Both synbiotic combinations increased lactate and all measured SCFAs; 2′-FL + M63 produced the highest acetate level, whereas 2′-FL + M16V produced the highest propionate level, and both combinations markedly increased butyrate. These findings demonstrate strain-dependent protective effects, with 2′-FL + M63 showing the greatest overall efficacy in restoring intestinal barrier function and microbial metabolism, supporting its development as a candidate functional synbiotic food formulation.

## 1. Introduction

Inflammatory bowel disease (IBD) comprises a group of disorders characterized primarily by chronic and recurrent intestinal inflammation, including ulcerative colitis and Crohn’s disease. In recent years, the global prevalence of IBD has continued to increase, with its burden gradually extending from Western countries to newly industrialized regions, making it a major public health concern [1]. The onset and progression of colitis are influenced by multiple factors, including genetic susceptibility, environmental factors, gut-microbiota dysbiosis, intestinal epithelial barrier disruption, and immune dysregulation. Among these factors, excessive release of pro-inflammatory cytokines, mucus-layer impairment, reduced expression of tight-junction proteins, and gut-microbiota metabolic abnormalities are considered key mechanisms underlying persistent inflammation [2,3]. Current treatment strategies for colitis include 5-aminosalicylic acid-based drugs, glucocorticoids, immunomodulators, biologics, and dietary interventions. However, long-term pharmacological therapy remains limited by interindividual variability in efficacy, high relapse rates, substantial costs, and potential adverse effects [4]. Therefore, the development of safe, mild, and sustainable nutritional interventions is of considerable significance. Probiotics, prebiotics, and their synbiotic combinations may promote intestinal health by modulating the gut microbiota and immune response, strengthening the intestinal barrier, and increasing short-chain fatty acid production [5,6].

2′-Fucosyllactose (2′-FL) is one of the most abundant fucosylated oligosaccharides in human milk. It has been approved for use in infant formula in regions and countries such as the United States, Europe, and China [7]. Previous studies have shown that 2′-FL can attenuate body weight loss and colon shortening in dextran sulfate sodium (DSS)-induced colitis models, inhibit the release of pro-inflammatory cytokines such as IL-1β, IL-6, and TNF-α, promote goblet cell recovery and MUC2 secretion [8], and ameliorate intestinal inflammation by regulating the gut microbiota and related metabolic pathways [9]. *Bifidobacterium* are an important type of beneficial bacteria in the gut of infants and young children. 2′-FL acts as a selective substrate for HMO-utilizing *Bifidobacterium* strains, particularly B. infantis M63, which can efficiently metabolize this oligosaccharide [10]. By contrast, *B. breve* M16V has been reported to possess immunomodulatory and anti-inflammatory potential and may participate in microbial interactions under specific oligosaccharide conditions [11]. *B. infantis* M63 and *B. breve* M16V are the two *Bifidobacterium* strains most widely used in food products, particularly in infant formula, milk powder for children, and probiotic dietary supplements [12]. However, the capacity to utilize 2′-FL varies among different *Bifidobacterium* species [6]. Furthermore, systematic evidence remains limited regarding whether 2′-FL combined with specific *Bifidobacterium* strains, particularly *B. infantis* M63 and *B. breve* M16V, produces strain-dependent synergistic protective effects. Therefore, the combination of 2′-FL with *Bifidobacterium* represents a promising strategy for the development of functional foods and nutritional supplements.

Therefore, this study used a dextran sulfate sodium (DSS)-induced acute colitis mouse model to systematically evaluate the therapeutic effects of 2′-FL alone and in combination with either *B. infantis* M63 or *B. breve* M16V. The effects of the synbiotic combinations were evaluated by assessing body weight, spleen index, colon length, hematoxylin and eosin (HE) staining, Alcian blue–periodic acid–Schiff (AB-PAS) staining, cytokine levels, oxidative stress, and intestinal-barrier function. In addition, the effects of the synbiotic combinations on gut microbiota composition, fermentation capacity, and metabolic function were analyzed by profiling the gut microbiota and measuring intestinal lactate and short-chain fatty acids (SCFAs). This study aimed to clarify strain-dependent differences in the efficacy of 2′-FL combined with different *Bifidobacterium* strains in alleviating DSS-induced colitis, thereby providing a theoretical basis and experimental support for developing functional foods or nutritional intervention products based on 2′-FL and *Bifidobacterium*.

## 2. Materials and Methods

### 2.1. Sources of Strains and Major Experimental Materials

Direct Vat Set (DVS) cultures of *B. infantis* M63 and *B. breve* M16V were obtained from Morinaga Milk Industry Co., Ltd. (Tokyo, Japan). 2′-fucosyllactose (2′-FL, ≥98% purity) was obtained from DSM (Heerlen, Netherlands). Dextran sulfate sodium (DSS, MW 36–50 kDa) was purchased from MP Biomedicals (Solon, OH, USA). TNF-α, Interleukin 1β (IL-1β), IL-6, IL-10, CAT, GSH-Px and MDA enzyme immunoassay kits were supplied by Youke Life Sciences Co., Ltd. (Hangzhou, China). *Claudin-1*, *Claudin-2*, *Occludin*, *ZO-1*, *RELMβ*-1, TFF-3 and MUC2 primers were supplied by Sangong Bioengineering Co., Ltd. (Shanghai, China).

### 2.2. Mouse Grouping, Housing, and Intervention

Forty-eight 6-week-old male C57BL/6J mice were purchased from Beijing Vital River Laboratory Animal Technology Co., Ltd. (Beijing, China). The mice were housed under controlled conditions at 25 ± 2 °C, 45 ± 5% relative humidity, and a 12-h light/dark cycle. After 1 week of acclimatization, the mice were randomly divided into six groups: negative control (NC), model control (MC), positive control (PC), 2′-FL intervention group (2′-FL), 2′-FL + *B. infantis* M63 intervention group (2′-FL + M63), and 2′-FL + *B. breve* M16V intervention group (2′-FL + M16V). Each group contained eight mice, with two mice housed per cage. The experimental intervention lasted 2 weeks. The sources of the intervention substances and DSS solution are shown in Table 1. Lactose (100 mg/mL) and 2′-FL (100 mg/mL) were administered at 400 mg/kg body weight (BW), *B. infantis* M63 and *B. breve* M16V were administered at 5 × 10^9^ CFU/kg BW, and 5-aminosalicylic acid (25 mg/mL) was administered at 100 mg/kg BW [13]. The intragastric dosage of 2′-FL was calculated using the body surface area (BSA) method, while the strain concentration was based on previously reported data [14]. All intervention substances were administered via gavage once daily. The bacteria (*B. infantis* M63 and *B. breve* M16V) were cultured anaerobically in MRS medium at 37 °C for 24 h and subcultured three times. The bacterial suspension was centrifuged at 8000× *g* for 15 min and subsequently resuspended in 10% skim milk. The strain concentration for oral administration was determined by viable cell counting prior to the experiment. Dissolve 2.5 g of DSS in 100 mL of water to prepare a 2.5% (*m*/*v*) DSS solution, and replace it once daily. On the final day of the experiment, after 12 h of fasting with free access to water, blood was collected from the orbital venous plexus. The mice were anesthetized with carbon dioxide and euthanized. The mice were dissected, and samples—including serum, colon, and spleen—were collected and stored at −80 °C for subsequent analysis.

### 2.3. Measurement of Weight-Gain Rate, Spleen Index, and Colon Length

Starting from the first day of the second week, the weight of each mouse was measured daily, with data from 8 mice in each group recorded as replicates. The rate of change in body weight was calculated (Formula (1)). Furthermore, the changes in the mice’s body weight were not statistically analyzed. At the end of the experiment, spleens were collected, and the spleen index (Formula (2)) was calculated as the ratio of spleen weight to body weight. Colons were collected, and their lengths were measured.

(1) $$weight\;change\;rate\;(\%)=\frac{body\;weight\;on\;day\;t}{body\;weight\;at\;baseline}\times 100$$

(Formula (1), “Body weight at baseline” refers to the first day of the second week of the experiment.)

(2) $$Spleen\;index\;(mg/g)=\frac{spleen\;weight\;(mg)}{final\;body\;weight\;(g)}\times 100$$

### 2.4. Hematoxylin and Eosin Staining

Colon tissues were rinsed with prechilled phosphate-buffered saline (PBS), fixed in 4% paraformaldehyde, dehydrated through graded ethanol, embedded in paraffin, and cut into 2–3-µm-thick sections. The sections were deparaffinized, stained with hematoxylin and eosin (HE), dehydrated, cleared, and mounted. For each animal, 3 non-consecutive separated sections were evaluated. From each section, non-overlapping microscopic fields were examined at 200 magnification using a light microscope (Leica DM5000B, Leica Microsystems, Wetzlar, Germany).

Histological evaluation was independently performed by 3 investigators blinded to the experimental-group allocation. Each field was scored for four criteria: (1) epithelial damage: 0, intact epithelium; 1, focal epithelial lifting or erosion; 2, extensive epithelial erosion; and 3, ulceration; (2) crypt architecture: 0, normal crypts; 1, mild crypt distortion or loss of ≤one-third of crypts; 2, loss of one-third to two-thirds of crypts; and 3, loss of >two-thirds of crypts or complete crypt loss; (3) inflammatory-cell infiltration: 0, absent; 1, mild infiltration confined to the mucosa; 2, moderate infiltration extending into the submucosa; and 3, severe or transmural infiltration; and (4) mucosal integrity: 0, intact mucosa; 1, focal mucosal disruption; 2, multifocal or continuous mucosal disruption; and 3, extensive ulceration and overall tissue destruction. Pathological changes in mouse intestinal tissue were evaluated based on the four criteria mentioned above, using scores assigned independently by researchers.

### 2.5. Alcian Blue Periodic Acid Schiff Staining

Paraffin-embedded colon tissues were cut into 4-µm-thick sections and stained with Alcian blue–periodic acid–Schiff (AB-PAS). For each animal, 3 non-consecutive separated sections were evaluated. Histological images were acquired using a VS200 digital pathology scanner (Olympus, Tokyo, Japan) at 200× magnification. The continuity of the colonic mucosa and mucus layer, crypt organization, and the distribution and staining intensity of AB-PAS-positive goblet cells and mucus were evaluated qualitatively by 3 investigators blinded to the experimental-group allocation.

### 2.6. Determination of Cytokine Levels in Colonic Tissues

After weighing 50 mg of colon tissue, the sample was mixed with ice-cold PBS (pH 7.4) and protease inhibitors, then homogenized. The homogenate was centrifuged at 5000× *g* for 10 min, and the supernatant was collected for subsequent analysis. Levels of tumor necrosis factor-α (TNF-α), interleukin-1β (IL-1β), IL-6, and IL-10 were quantitatively measured according to the manufacturer’s instructions for the enzyme-linked immunosorbent assay (ELISA) kits (Youke Life Sciences Co., Ltd. Hangzhou, China). The total protein concentration in the supernatant was determined using the BCA assay, and cytokine levels were calculated using Equation (3):

(3) $$Cytokine\;level\;(pg/mg\;protein)=\frac{Measured\;cytokine\;concentration\;(pg/mL)}{Total\;protein\;concentration\;(mg/mL)}$$

### 2.7. Assessment of Oxidative Stress in Colonic Tissues

The activities of catalase (CAT) and glutathione peroxidase (GSH-Px) and the level of malondialdehyde (MDA) were determined using ELISA kits. Sample pretreatment, kit operation, and concentration calculations were performed as described in Section 2.6.

### 2.8. RT-qPCR Analysis of Tight-Junction- and Mucus-Barrier-Associated Gene Expression

Colon tissues were collected, and total RNA was extracted using TRIzol reagent according to the manufacturer’s protocol. RNA concentration and purity were determined spectrophotometrically, and only RNA samples with A260/A280 ratios of 1.8–2.0 were used for subsequent analysis. Reverse transcription was performed using 3 µg of total RNA and the FastKing RT Kit (Tiangen Biotech, Beijing, China) according to the manufacturer’s instructions. Quantitative PCR was conducted using the SYBR Green Premix Pro Taq HS qPCR Kit (Accurate Biotechnology, Changsha, China) on a QuantStudio 5 Real-Time PCR System (Applied Biosystems, Foster City, CA, USA). Each 20 µL reaction contained 10 µL of 2× SYBR Green Premix Pro Taq HS, 0.4 µL of forward primer (10 µM), 0.4 µL of reverse primer (10 µM), 2 µL of cDNA template, and 7.2 µL of nuclease-free water; the final concentration of each primer was 0.2 µM. The thermal cycling program consisted of initial denaturation at 95 °C for 10 min, followed by 40 cycles of denaturation at 95 °C for 15 s and annealing/extension at 60 °C for 1 min. A dissociation-curve stage of 95 °C for 15 s, 60 °C for 15 s, and 95 °C for 15 s was subsequently performed to assess amplification specificity.

Each cDNA sample was analyzed in 3 technical replicates, and the mean threshold-cycle value was used for calculation. The threshold cycle (Ct) was defined as the cycle number at which the fluorescence signal crossed the predefined threshold. GAPDH was used as the reference gene, and the negative control (NC) group was used as the calibrator. Relative gene expression was calculated using the 2^−ΔΔCt^ method as follows:

(4) ∆Ct=Ct target gene−Ct reference gene

(5) ∆∆Ct=∆Ct treatment−∆Ct calibrat

Relative gene expression = 2^−ΔΔCT^ (6)

where Ct_target gene, sample and Ct_GAPDH, sample are the Ct values of the target gene and GAPDH, respectively; ΔCt_sample is the target-gene Ct normalized to GAPDH; and ΔΔCt_sample is the ΔCt of an individual sample relative to the mean ΔCt of the NC calibrator group. The relative expression level of the NC group was therefore set to 1. Primer sequences are listed in Table 2. GAPDH was selected as the internal reference gene based on previous studies [15].

### 2.9. Gut Microbiome Analysis

Fecal samples were collected from mice on day 7, and intestinal contents were collected on day 14. On day 7, considering the need to continue feeding the mice, mouse feces were selected for gut-microbiome analysis. Due to the varying quality of the collected feces, the NC group ultimately underwent 5 parallel experiments, while the other groups underwent 8 parallel experiments. Additionally, on day 14, all mice were controlled for 3 replicates. Sample selection involved randomly selecting 3 mice from each group of 8 for collecting gut contents for microbiome analysis.

Microbial genomic DNA was extracted using the cetyltrimethylammonium bromide (CTAB) method, and DNA integrity was assessed by agarose gel electrophoresis. PCR amplification was performed using diluted genomic DNA as the template. The V3–V4 region of the bacterial 16S rRNA gene was amplified using primers 338F 5′-ACTCCTACGGGAGGCAGCA-3′ and 806R 5′-GGACTACHVGGGTWTCTAAT-3′. Sequencing libraries were constructed using the TruSeq^®^ DNA PCR-Free Sample Preparation Kit (Illumina, San Diego, CA, USA). The constructed libraries were quantified using Qubit and quantitative PCR (qPCR), and sequencing was performed on the NovaSeq 6000 platform. The PCR reaction was performed in a total volume of 50 µL, containing 5 µL of 10× KOD buffer, 5 µL of 2.5 mM dNTPs, 1.5 µL of each primer (5 µM; final concentration of 0.15 µM), 1 µL of KOD DNA polymerase, 100 ng of template DNA, and nuclease-free water. The amplification protocol consisted of an initial denaturation at 95 °C for 2 min, followed by 27 cycles of denaturation at 98 °C for 10 s, annealing at 62 °C for 30 s, and extension at 68 °C for 30 s, with a final extension at 68 °C for 10 min. A no-template control was included in each PCR run. Amplification products were verified by 2% agarose gel electrophoresis; products showing a single band of the expected size were purified using the AxyPrep DNA Gel Extraction Kit (Axygen Biosciences, Union City, CA, USA) and quantified using a fluorescence-based method.

Demultiplexing and removal of sample barcodes, adapter sequences, and primer sequences were conducted before quality filtering. Reads containing more than 10% ambiguous nucleotides or reads in which fewer than 80% of bases had a Phred quality score greater than 20 were discarded. Paired-end reads were merged using FLASH v1.2.11 with a minimum overlap of 10 bp and a maximum mismatch rate of 2%; reads that could not be merged were removed. Additional quality filtering was performed using QIIME v1.9.1. Chimeric sequences were detected and removed within the UPARSE pipeline. The sequences retained after all quality-control steps were defined as effective tags. Effective tags were clustered into operational taxonomic units (OTUs) at 97% sequence identity using UPARSE v7.0.1001, and the most abundant sequence in each OTU was selected as the representative sequence. Alpha- and beta-diversity analyses were performed in QIIME v1.9.1. LEfSe v1.1.2 was used to identify differential taxa.

Alpha diversity was evaluated using the Chao1 richness and Shannon diversity indices. Differences among groups were tested using the Kruskal–Wallis test followed by Dunn’s post hoc test. Beta diversity was calculated using unweighted UniFrac distances and visualized by principal component analysis (PCA). Group-level differences in community composition were evaluated by PERMANOVA using the Adonis function with 999 permutations. Relative abundances at the phylum and genus levels were compared using the Kruskal–Wallis test followed by Dunn’s test. For alpha-diversity and taxon-level multiple comparisons, *p* values were adjusted using the Benjamini–Hochberg false-discovery-rate procedure, and q < 0.05 was considered significant. LEfSe analysis used a Kruskal–Wallis significance threshold of *p* < 0.05 and an LDA score threshold of 2.0; LEfSe *p* values were not additionally adjusted.

The raw sequencing data are available at Mendeley Data, doi: 10.17632/7fdt7t8vvw.1.

### 2.10. Determination of Short-Chain Fatty Acids and Lactic Acid

Acetate, propionate, and butyrate were quantified by gas chromatography with flame-ionization detection (GC-FID), whereas lactate was determined using a separate pre-column-derivatization high-performance liquid chromatography (HPLC). Analytical standards of acetic acid, propionic acid, butyric acid, and lactic acid were calculated (purity ≥ 99%; Shanghai Yuanye Bio-Technology Co., Ltd., Shanghai, China). The initial concentration of the four standards was 10 mmol/L. The mixed standard solution was prepared fresh before use. Standard curves for the four standards were constructed using five concentration levels (25, 50, 100, 200, and 400 µmol/L). A 0.7 g sample of intestinal contents collected on day 14 was weighed. Then, 1.0 mL of 6% phosphoric acid solution (V_0_) was added to a 2 mL homogenization tube containing 3-mm stainless steel beads, and the sample was homogenized under refrigerated conditions at 50 Hz for 2 min. The mixture was vortexed, the stainless steel beads were removed, and the sample was centrifuged at 10,000× *g* for 15 min. The supernatant was then filtered through a 0.22-µm syringe filter into a sample vial for instrumental analysis.

The chromatographic conditions for GC-FID were as follows: injector temperature, 220 °C; injection volume, 1 µL; and split ratio, 50:1. High-purity nitrogen (N_2_) was used as the carrier gas at a flow rate of 1 mL/min. The column was a DB-FAXWAX (30 m × 0.25 mm × 0.25 µm, Agilent Technologies, Santa Clara, CA, USA). The oven temperature program was as follows: initial temperature 80 °C, held for 1.5 min; increased to 125 °C at 45 °C/min and held for 2.5 min; then increased to 170 °C at 40 °C/min; and finally increased to 225 °C at 80 °C/min and held for 1 min. Flame ionization detector (FID) temperature was set at 250 °C. Hydrogen flow rate was 40 mL/min, zero-air flow rate was 450 mL/min, and nitrogen purge flow rate was 35 mL/min.

The HPLC chromatographic conditions were as follows: lactic acid was derivatized with aniline. A CRS10 W column (4.6 mm × 50 mm, 3 μm) was selected. The mobile phase was 2 mmol/L CuSO_4_·5H_2_O aqueous solution, the flow rate was 0.5 mL/min, the column temperature was 25 °C, and the injection volume was 10 μL.

Chromatographic peaks were identified by comparing retention times with those of standards, and the four components were quantified using the external standard method. The standard curves for the four standards are as follows: acetic acid: y = 2723.8x + 2789.2 (R^2^ = 0.9915); propionic acid: y = 6322.4x − 589.2 (R^2^ = 0.9937); butyric acid: y = 9021.0x − 1892.5 (R^2^ = 0.9901); lactic acid: y = 5718.6x − 672.3 (R^2^ = 0.9951).

The formula for calculating organic acids in the sample is as follows:

(7) $$Organic\;acid\;content=\frac{C\times V\times 200}{m}$$

where *C* is the concentration of organic acid in the extract to be tested (µmol/L) obtained according to the corresponding calibration curve, *V* is the total extraction volume (mL), *m* is the mass (g) of the intestinal contents sample used for extraction, and 200 is the dilution factor.

### 2.11. Statistical Analysis

Statistical analyses were performed using IBM SPSS Statistics version 26.0 (IBM Corp., Armonk, NY, USA). Samples from at least three animals were selected for analysis in each experiment. The biological sample sizes were eight animals per group for longitudinal body-weight analysis; six animals per group for spleen index, colon length, and RT-qPCR; four animals per group for cytokine, oxidative-stress, lactate, and short-chain fatty acid analyses; three animals per group for the day-14 gut-microbiota analysis; and five to eight animals per group for the day-7 gut-microbiota analysis.

For single-time-point continuous outcomes, normality of residuals was assessed using the Shapiro–Wilk test, and homogeneity of variances was assessed using Levene’s test. Data satisfying both assumptions were compared among groups by one-way analysis of variance (ANOVA), followed by Tukey’s honestly significant difference test for all pairwise comparisons. When normality was satisfied but variances were unequal, Welch’s ANOVA followed by the Games–Howell test was used. Data that did not satisfy the normality assumption were analyzed using the Kruskal–Wallis test followed by Dunn’s multiple-comparison test with Benjamini–Hochberg adjustment. The statistical procedures used for alpha diversity, beta diversity, and taxonomic-abundance comparisons are described separately in Section 2.9. Based on statistical analysis, groups showing significant differences were marked with different letters, indicating statistical significance (*p* < 0.05).

## 3. Results

### 3.1. Synbiotic Combinations Alleviate Colitis-Related Phenotypes in Mice

**Figure 1:**
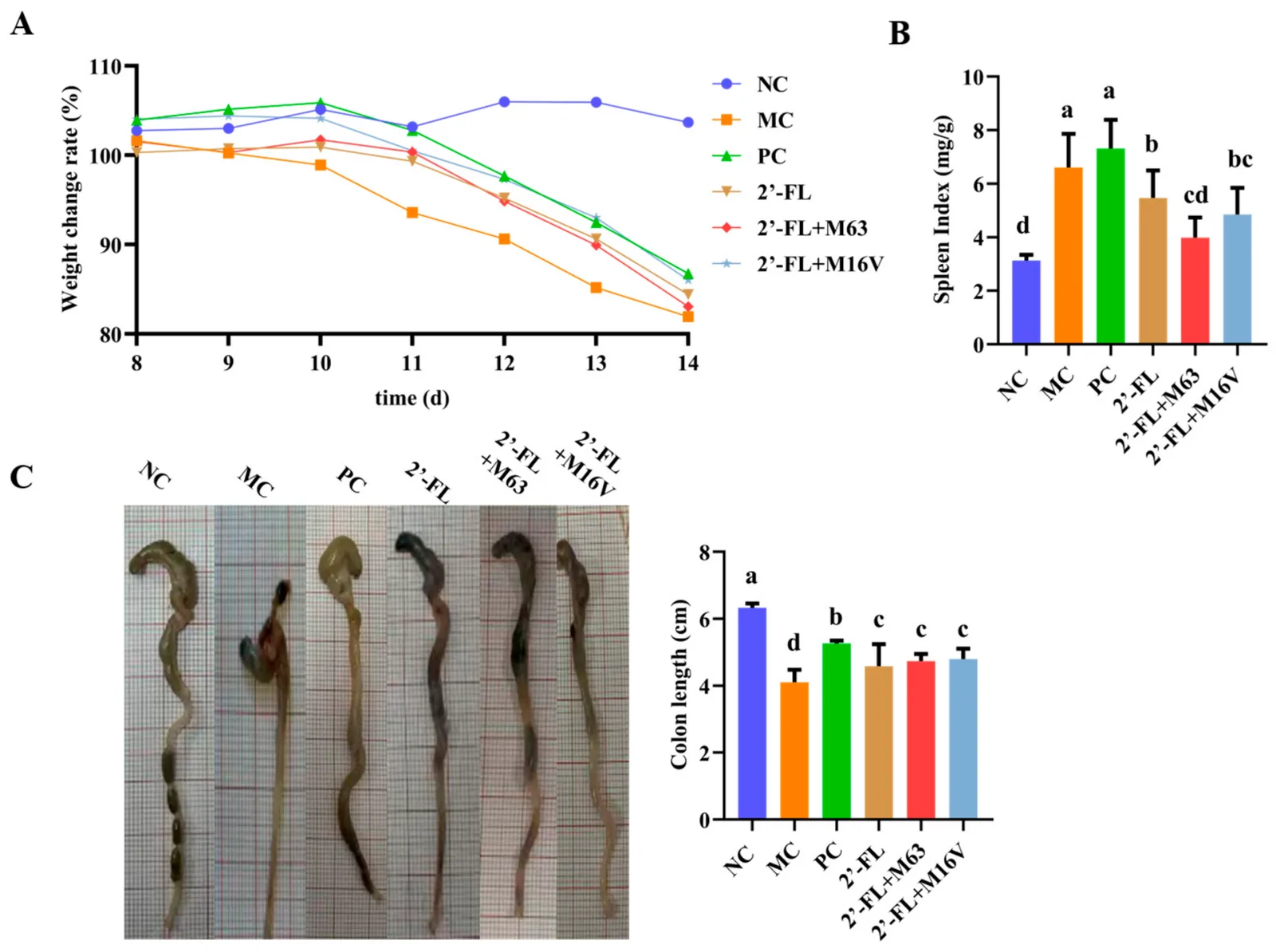
Synbiotics mitigate the effects of DSS on the spleen and colon in mice. (**A**) weight change rate (*n* = 8). (**B**) Spleen index (*n* = 6), Different letters (a–d) indicate statistically significant differences between groups (*p* < 0.05). (**C**) Schematic of the colon and intestinal measurements (*n* = 6). Negative control (NC), model control (MC), positive control (PC), 2′-FL intervention group (2′-FL), 2′-FL + B. infantis M63 intervention group (2′-FL + M63), and 2′-FL + B. breve M16V intervention group (2′-FL + M16V).

To evaluate the effects of 2′-FL alone and in combination with *Bifidobacterium* on DSS-induced colitis in mice, we assessed body weight, spleen index, colon length, and colonic histopathological features. DSS treatment induced marked colitis-related phenotypes in mice. Compared with the NC group, mice in the MC group showed marked body weight loss during the later stage of the experiment (Figure 1A), a significantly increased spleen index (Figure 1B, *p* < 0.05), and significantly shortened colon length (Figure 1C, *p* < 0.05), indicating successful establishment of the DSS-induced acute colitis model. Compared with the MC group, the treatments in the PC group, 2′-FL group, 2′-FL + M63 group and 2′-FL + M16V group had little effect on mouse body weight and colon length. In addition, 2′-FL alone and its combinations with *Bifidobacterium* significantly reduced the DSS-induced increase in spleen index (*p* < 0.05), with the 2′-FL + M63 group showing the most pronounced effect, suggesting a beneficial role in alleviating inflammation-related splenomegaly.

**Figure 2:**
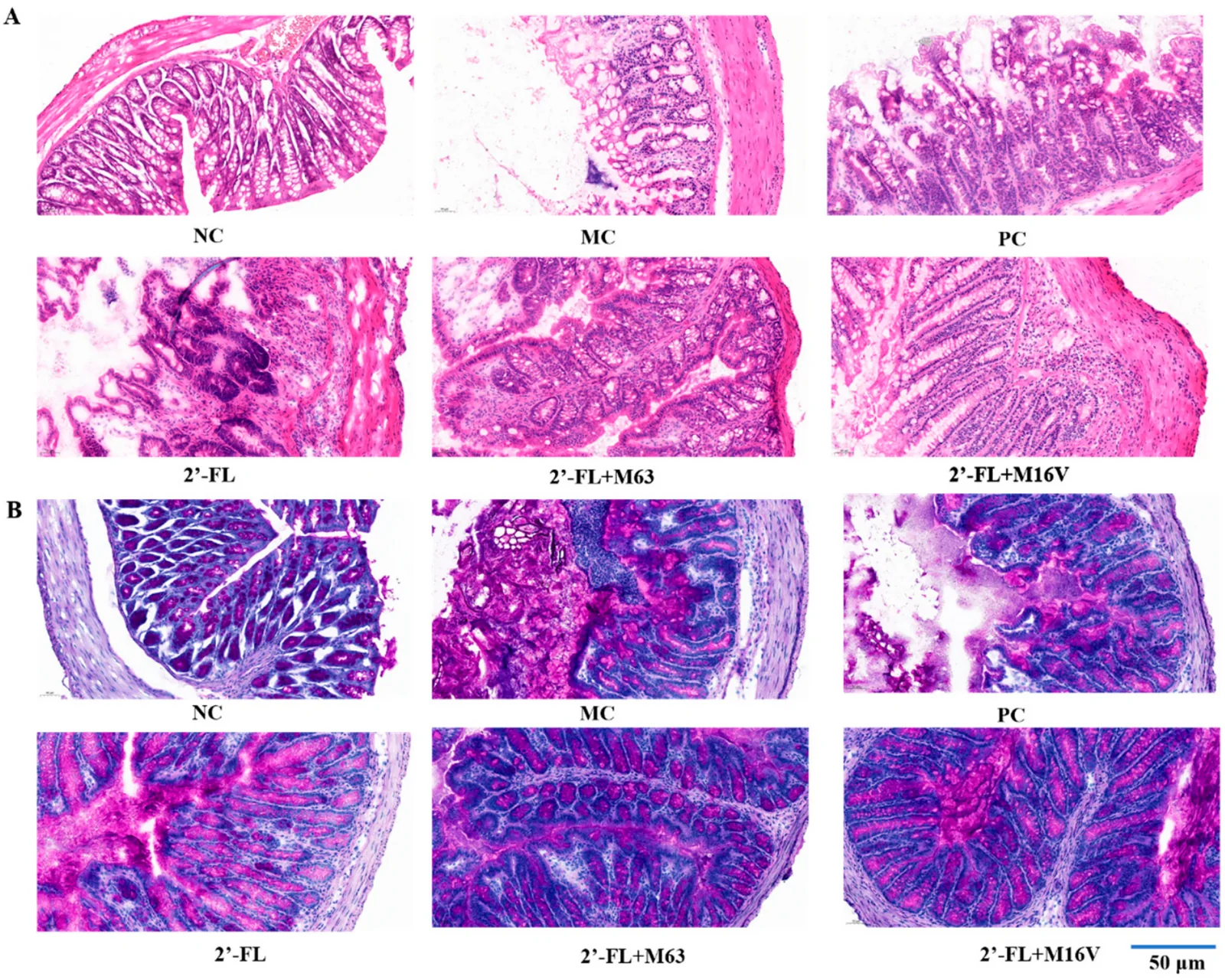
Synbiotics mitigate the effects of DSS on colonic pathology and goblet cells in mice. (**A**) Colon tissue pathology observed by HE staining. (**B**) Goblet cell function observed by AB-PAS staining. Negative control (NC), model control (MC), positive control (PC), 2′-FL intervention group (2′-FL), 2′-FL + B. infantis M63 intervention group (2′-FL + M63), and 2′-FL + B. breve M16V intervention group (2′-FL + M16V).

Subsequently, colonic histopathology was examined using hematoxylin and eosin (HE) and Alcian blue–periodic acid–Schiff (AB-PAS) staining. HE staining showed that the NC group had an intact colonic mucosal structure with regularly arranged crypts, whereas the MC group exhibited obvious epithelial damage, crypt disruption, and inflammatory cell infiltration (Figure 2A). Treatment with PC, 2′-FL, and both synbiotic combinations alleviated DSS-induced tissue damage, and the combination groups showed more intact mucosal structures than the MC group. AB-PAS staining showed that the MC group had significantly fewer goblet cells and weaker mucus-positive signals, whereas the 2′-FL, 2′-FL + M63, and 2′-FL + M16V groups promoted the recovery of goblet cells and the mucus layer; among these groups, the 2′-FL + M63 group showed goblet cell abundance and mucus structure most similar to those of the NC group (Figure 2B). In summary, 2′-FL combined with either *B. infantis* M63 or *B. breve* M16V effectively alleviated DSS-induced colitis-related phenotypes in mice, with the *B. infantis* M63 combination showing greater efficacy.

### 3.2. Synbiotic Combinations Reduce Intestinal Inflammation in Mice

**Figure 3:**
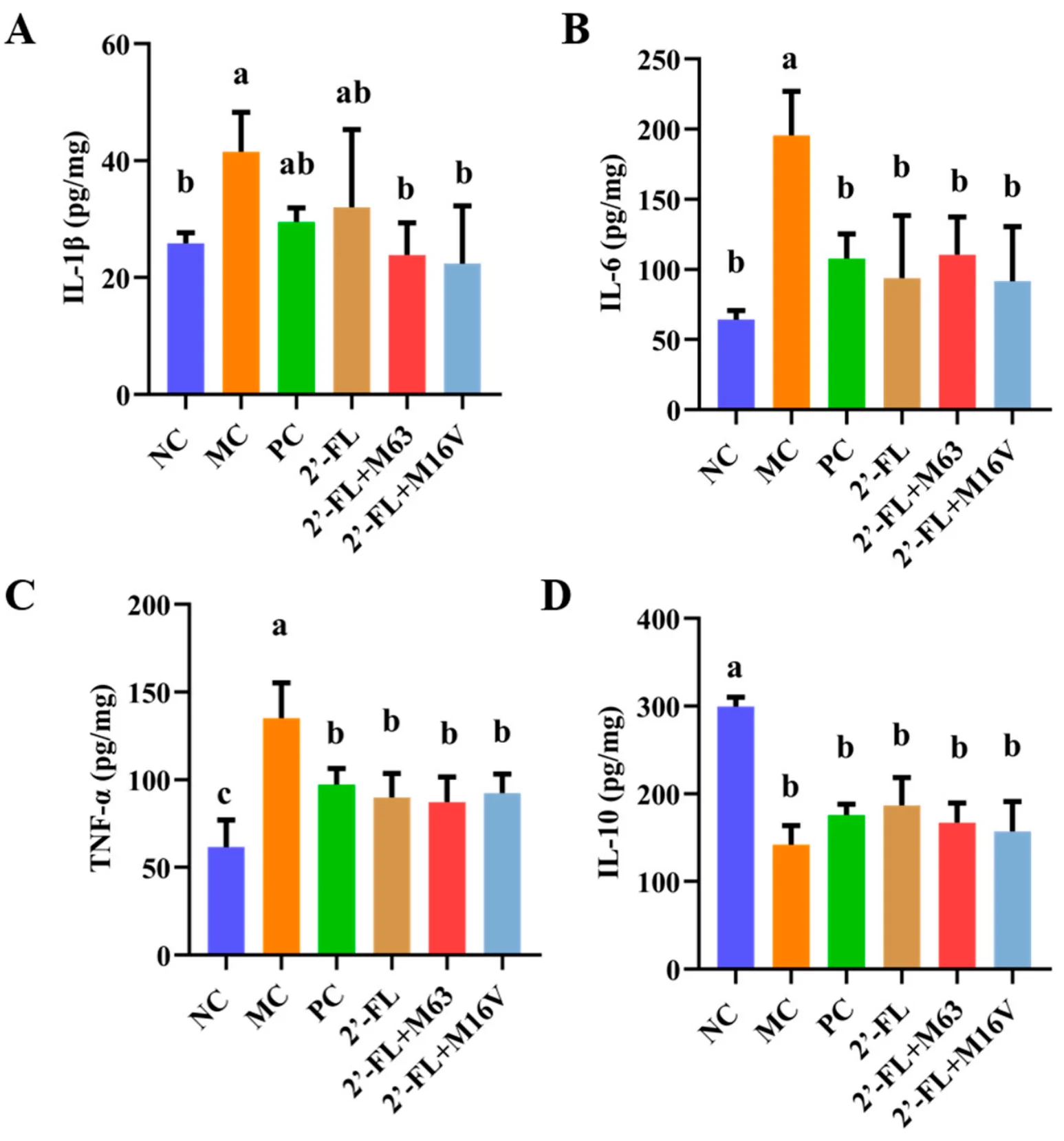
Synbiotics attenuate the inflammatory effects of DSS in mice. (**A**) IL-1β. (**B**) IL-6. (**C**) TNF-α. (**D**) IL-10. *n* = 4. Negative control (NC), model control (MC), positive control (PC), 2′-FL intervention group (2′-FL), 2′-FL + B. infantis M63 intervention group (2′-FL + M63), and 2′-FL + B. breve M16V intervention group (2′-FL + M16V). Note: Different letters (a–c) indicate statistically significant differences between groups (*p* < 0.05).

To evaluate the effects of the synbiotic combinations on DSS-induced intestinal inflammation, we measured the levels of the pro-inflammatory cytokines IL-1β, IL-6, and TNF-α, as well as the anti-inflammatory cytokine IL-10, in colonic tissues. As shown in Figure 3A–D, compared with the NC group, the MC group exhibited significantly increased levels of IL-1β, IL-6, and TNF-α (*p* < 0.05) and significantly decreased levels of IL-10 (*p* < 0.05), indicating that DSS treatment induced a marked colonic inflammatory response. Compared with the MC group, the 2′-FL + M63 and 2′-FL + M16V groups showed significantly reduced IL-1β levels (*p* < 0.05). The PC, 2′-FL, and both synbiotic combination groups showed significantly reduced IL-6 and TNF-α levels (*p* < 0.05). Although IL-10 levels in the intervention groups showed a trend toward recovery, the differences were not significant compared with the MC group (*p* > 0.05). These results indicate that 2′-FL, either alone or in combination with *B. infantis* M63 or *B. breve* M16V, alleviates DSS-induced intestinal inflammation in mice primarily by suppressing excessive pro-inflammatory cytokine production.

### 3.3. Synbiotic Combinations Suppress Colitis-Associated Intestinal Oxidative Stress

**Figure 4:**
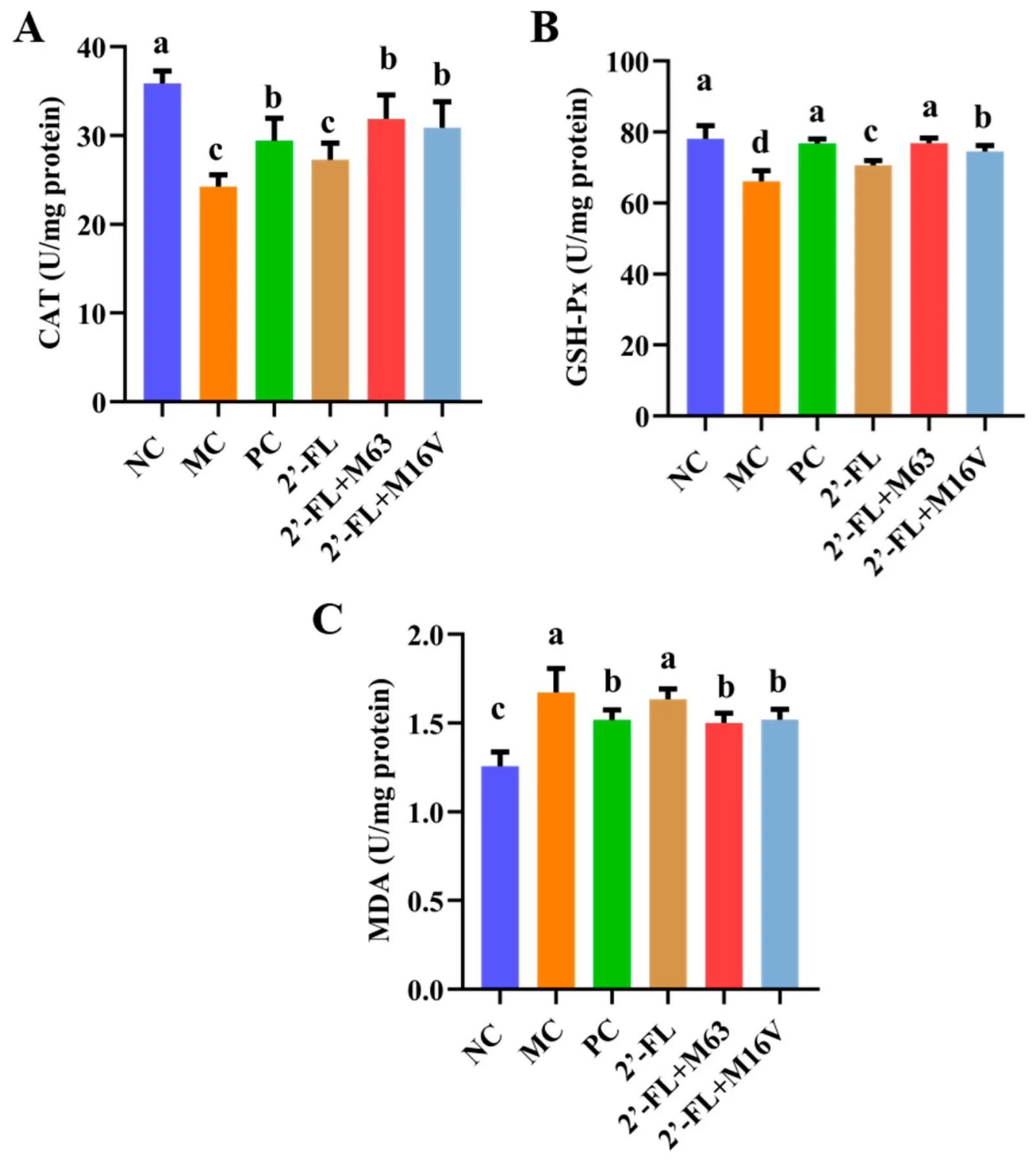
Synbiotics mitigate the effects of DSS on intestinal oxidative stress in mice. (**A**) CAT. (**B**) GSH-Px. (**C**) MDA. *n* = 4. Negative control (NC), model control (MC), positive control (PC), 2′-FL intervention group (2′-FL), 2′-FL + B. infantis M63 intervention group (2′-FL + M63), and 2′-FL + B. breve M16V intervention group (2′-FL + M16V). Note: Different letters (a–d) indicate statistically significant differences between groups (*p* < 0.05).

Because inflammatory responses are often accompanied by increased oxidative stress, oxidative stress-related markers, including catalase (CAT), glutathione peroxidase (GSH-Px), and malondialdehyde (MDA), were further measured in colonic tissues. As shown in Figure 4A–C, compared with the NC group, the MC group exhibited significantly decreased CAT and GSH-Px activities and significantly increased MDA levels (*p* < 0.05), indicating that DSS-induced colitis was accompanied by substantial oxidative stress. Compared with the MC group, the PC, 2′-FL + M63, and 2′-FL + M16V groups showed significantly increased CAT activity (*p* < 0.05), with the two synbiotic combination groups showing a more pronounced restorative effect (Figure 4A). CAT is an important antioxidant enzyme that decomposes excess hydrogen peroxide and mitigates reactive oxygen species (ROS)-induced damage. Moreover, the PC, 2′-FL, 2′-FL + M63, and 2′-FL + M16V groups all showed significantly increased GSH-Px activity (*p* < 0.05), with the 2′-FL + M63 group approaching the level observed in the NC group (Figure 4B). GSH-Px catalyzes the glutathione-dependent reduction in lipid peroxides and is a key enzyme involved in maintaining cellular redox homeostasis. Furthermore, MDA is an end product of lipid peroxidation, and elevated MDA levels typically indicate increased oxidative tissue damage. MDA levels in the PC, 2′-FL + M63, and 2′-FL + M16V groups were significantly lower than those in the MC group (*p* < 0.05), whereas 2′-FL alone did not significantly reduce MDA levels (Figure 4C). These results indicate that *Bifidobacterium* enhances the effects of 2′-FL in boosting antioxidant enzyme activity and inhibiting lipid peroxidation, thereby alleviating DSS-induced intestinal oxidative stress.

### 3.4. Synbiotic Combinations Modulate Tight-Junction-Associated Gene Expression in Colonic Tissue

**Figure 5:**
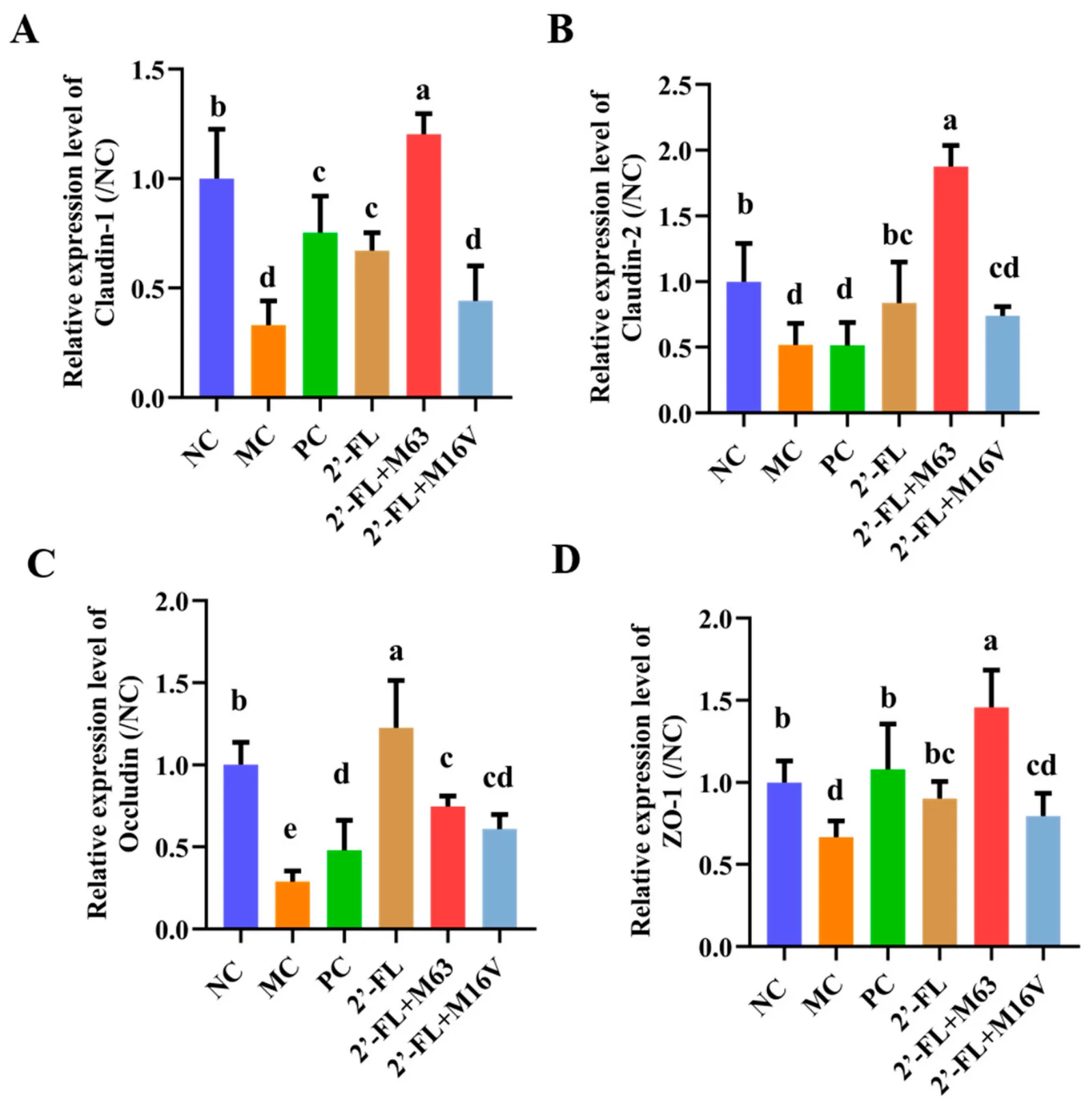
Synbiotics regulate the expression of tight junctions in the mouse intestine. (**A**) *Claudin-1*. (**B**) *Claudin-2*. (**C**) *Occludin*. (**D**) *ZO-1*. *n* = 6. Negative control (NC), model control (MC), positive control (PC), 2′-FL intervention group (2′-FL), 2′-FL + B. infantis M63 intervention group (2′-FL + M63), and 2′-FL + B. breve M16V intervention group (2′-FL + M16V). Note: Different letters (a–e) indicate statistically significant differences between groups (*p* < 0.05).

To evaluate treatment-associated molecular changes relevant to the epithelial junctional barrier, we measured the relative mRNA expression levels of the tight-junction-associated genes *Claudin-1*, *Claudin-2*, *Occludin*, and *ZO-1* in colonic tissues. As shown in Figure 5A–D, compared with the NC group, the MC group showed significantly lower relative mRNA expression levels of *Claudin-1*, *Claudin-2*, *Occludin*, and *ZO-1* (*p* < 0.05), indicating reduced transcription of several tight-junction-associated genes following DSS exposure. Compared with the MC group, the 2′-FL + M63 group showed significantly higher relative mRNA expression levels of *Claudin-1*, *Claudin-2*, and *ZO-1* (*p* < 0.05). These levels were also higher than those in the NC group (*p* < 0.05), indicating a marked treatment-associated transcriptional response rather than a simple return to the control baseline. 2′-FL alone significantly increased *Occludin* mRNA expression (*p* < 0.05). The PC group showed significantly higher *Claudin-1* and *ZO-1* mRNA expression than the MC group (*p* < 0.05), whereas the 2′-FL + M16V group showed comparatively limited effects on the measured transcripts. Overall, the 2′-FL-containing interventions differentially modulated tight-junction-associated gene expression, with the 2′-FL + M63 combination producing the broadest transcriptional response.

### 3.5. Synbiotic Combinations Promote the Expression of Mucus-Barrier-Associated Genes in the Intestine

**Figure 6:**
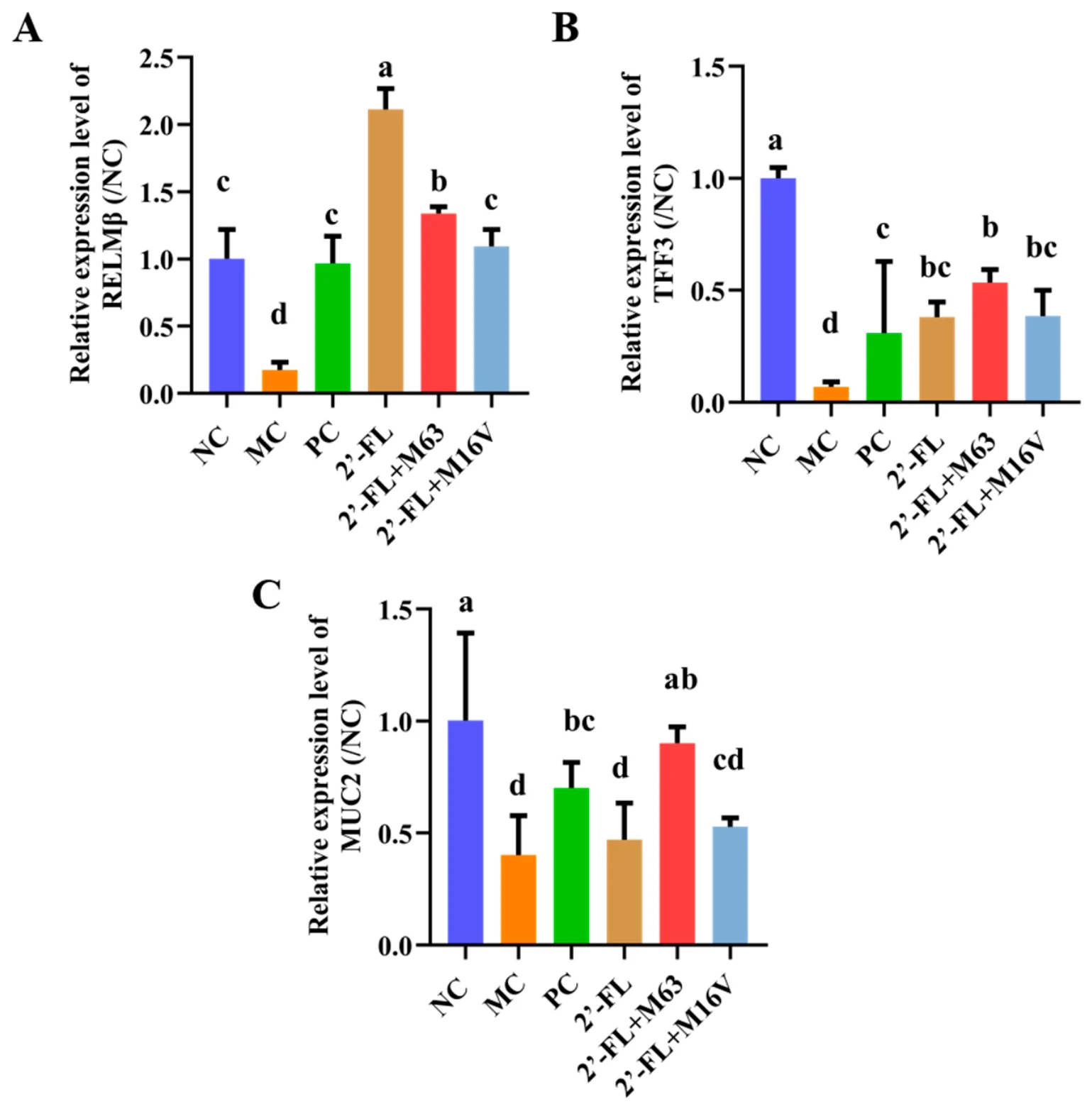
Synbiotics regulate the expression of mucin-related genes in the mouse intestine. (**A**) *RELMβ*-1. (**B**) TFF-3. (**C**) MUC2. *n* = 6. Negative control (NC), model control (MC), positive control (PC), 2′-FL intervention group (2′-FL), 2′-FL + B. infantis M63 intervention group (2′-FL + M63), and 2′-FL + B. breve M16V intervention group (2′-FL + M16V). Note: Different letters (a–d) indicate statistically significant differences between groups (*p* < 0.05).

MUC2 is the primary gel-forming mucin in the colonic mucus layer and is essential for maintaining mucus-barrier integrity at the intestinal epithelial surface. *TFF3* is primarily secreted by goblet cells and is involved in mucosal repair, epithelial restitution, and stabilization of the mucus layer. *RELMβ* is a mucin-associated protein secreted by intestinal goblet cells and is involved in host defense responses and mucosal homeostasis. As shown in Figure 6A–C, compared with the NC group, the MC group showed significantly reduced mRNA expression levels of *RELMβ*, *TFF3*, and MUC2 (*p* < 0.05), indicating that DSS-induced colitis impaired goblet cell secretory function and the mucus barrier. Compared with the MC group, the 2′-FL, 2′-FL + M63, and 2′-FL + M16V groups showed increased *RELMβ* mRNA expression to varying degrees, with the 2′-FL group showing the greatest increase, followed by the 2′-FL + M63 group. *TFF3* mRNA expression was also significantly restored in all treatment groups, with the 2′-FL + M63 group showing significantly higher levels than the PC, 2′-FL, and 2′-FL + M16V groups (*p* < 0.05), suggesting a stronger effect on mucosal repair-related factors. For MUC2, the 2′-FL + M63 group showed significantly higher mRNA expression than the MC group (*p* < 0.05) and levels comparable to those of the NC group (*p* > 0.05), whereas 2′-FL alone and the 2′-FL + M16V group showed relatively limited effects. Together with AB-PAS staining results, these findings indicate that synbiotic combinations, particularly 2′-FL combined with *B. infantis* M63, ameliorate DSS-induced colonic mucus-barrier damage by promoting mucus-barrier-associated gene expression.

### 3.6. Synbiotic Combinations Modulate Gut Microbiota Composition

**Figure 7:**
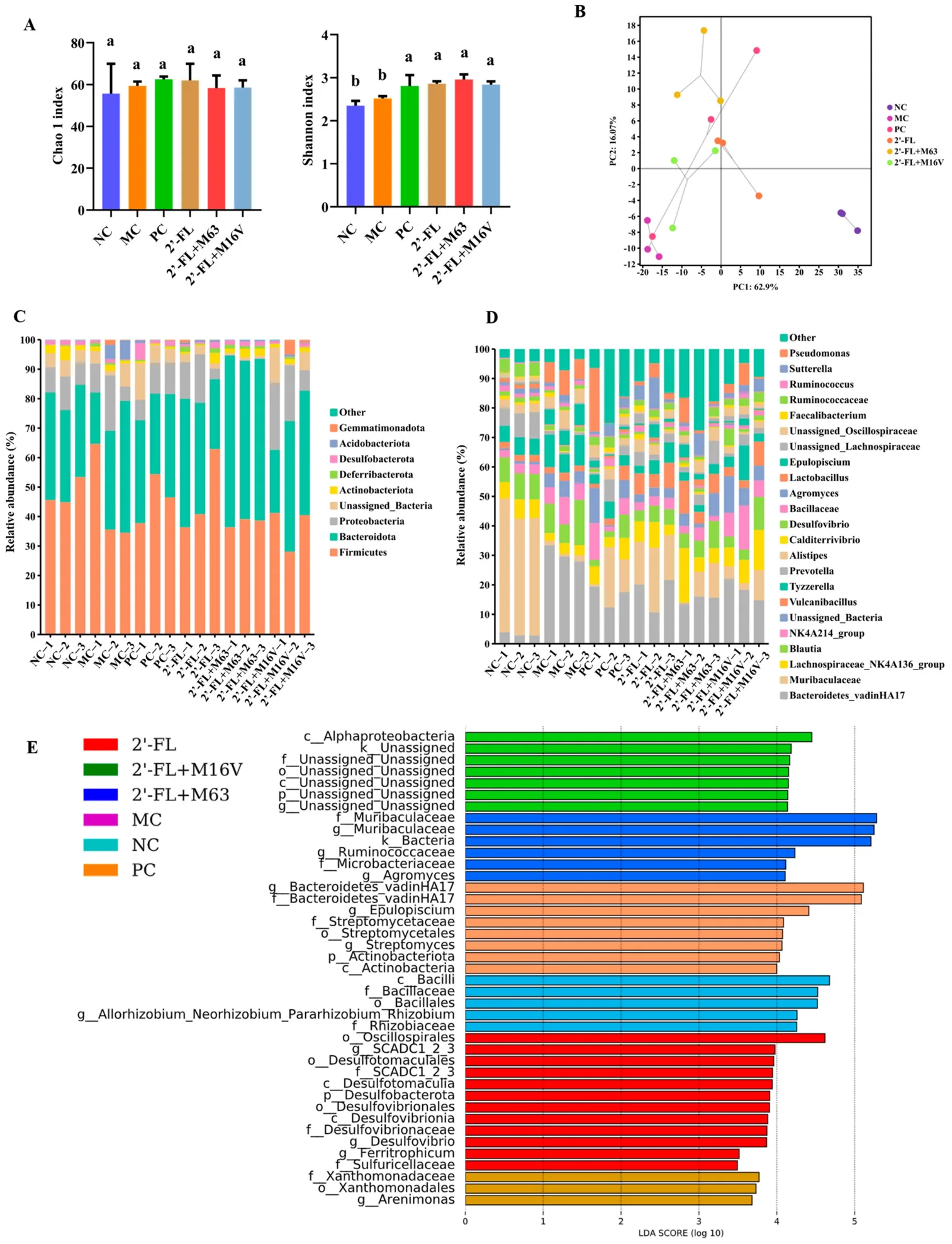
Synbiotics regulate the composition of the mouse gut microbiota. (**A**) α-diversity, including the Chao-1 index and Shannon index. (**B**) PCA. (**C**) Species composition at the phylum level. (**D**) Species composition at the genus level. (**E**) LEfSe analysis was used to identify biomarker bacterial taxa for each group. Negative control (NC), model control (MC), positive control (PC), 2′-FL intervention group (2′-FL), 2′-FL + B. infantis M63 intervention group (2′-FL + M63), and 2′-FL + B. breve M16V intervention group (2′-FL + M16V). Note: Different letters (a,b) indicate statistically significant differences between groups (*p* < 0.05).

The gut microbiota plays a crucial role in maintaining intestinal homeostasis, and DSS-induced colitis is typically accompanied by microbial dysbiosis. 2′-FL and *Bifidobacterium* are common microbiota modulators that confer health benefits by shaping the gut microbiota. Changes in gut microbiota composition following 2′-FL or synbiotic interventions were analyzed. The Chao1 and Shannon indices are metrics used to assess gut microbiota α-diversity. As shown in Figure 7A, no significant differences were observed in the Chao1 index among groups (*p* > 0.05). Compared with the MC group, the Shannon index was significantly higher in the 2′-FL, 2′-FL + M63, and 2′-FL + M16V groups (*p* < 0.05). These results indicate that 2′-FL or synbiotic interventions increased gut microbiota diversity. Furthermore, principal component analysis (PCA) revealed clear separation among groups and high within-group similarity (Figure 7B). At the phylum level, gut microbiota in each group was dominated by Firmicutes, Bacteroidota, and Proteobacteria (Figure 7C). Compared with the MC group, the 2′-FL + M63 group showed increased relative abundance of Bacteroidota and decreased relative abundance of Proteobacteria. At the genus level, dominant taxa included *Muribaculaceae*, *Bacteroides_vadinHA17_group*, *Lachnospiraceae_NK4A136_group*, *Blautia*, *Alistipes*, *Prevotella*, and *Lactobacillus* (Figure 7D). Compared with the MC group, 2′-FL and synbiotic interventions altered the relative abundances of several dominant genera. Notably, the 2′-FL + M63 group exhibited a higher abundance of *Muribaculaceae*-associated taxa, which are typically involved in polysaccharide and oligosaccharide utilization, suggesting that 2′-FL combined with *B. infantis* M63 may promote the proliferation of carbohydrate-utilizing microbiota. Linear discriminant analysis effect size (LEfSe) analysis was used to identify discriminative taxonomic biomarkers under different treatments. As shown in Figure 7E, the 2′-FL + M63 group was enriched in *Muribaculaceae* and *Ruminococcaceae*, whereas the 2′-FL + M16V group was enriched in *Alphaproteobacteria*.

**Figure 8:**
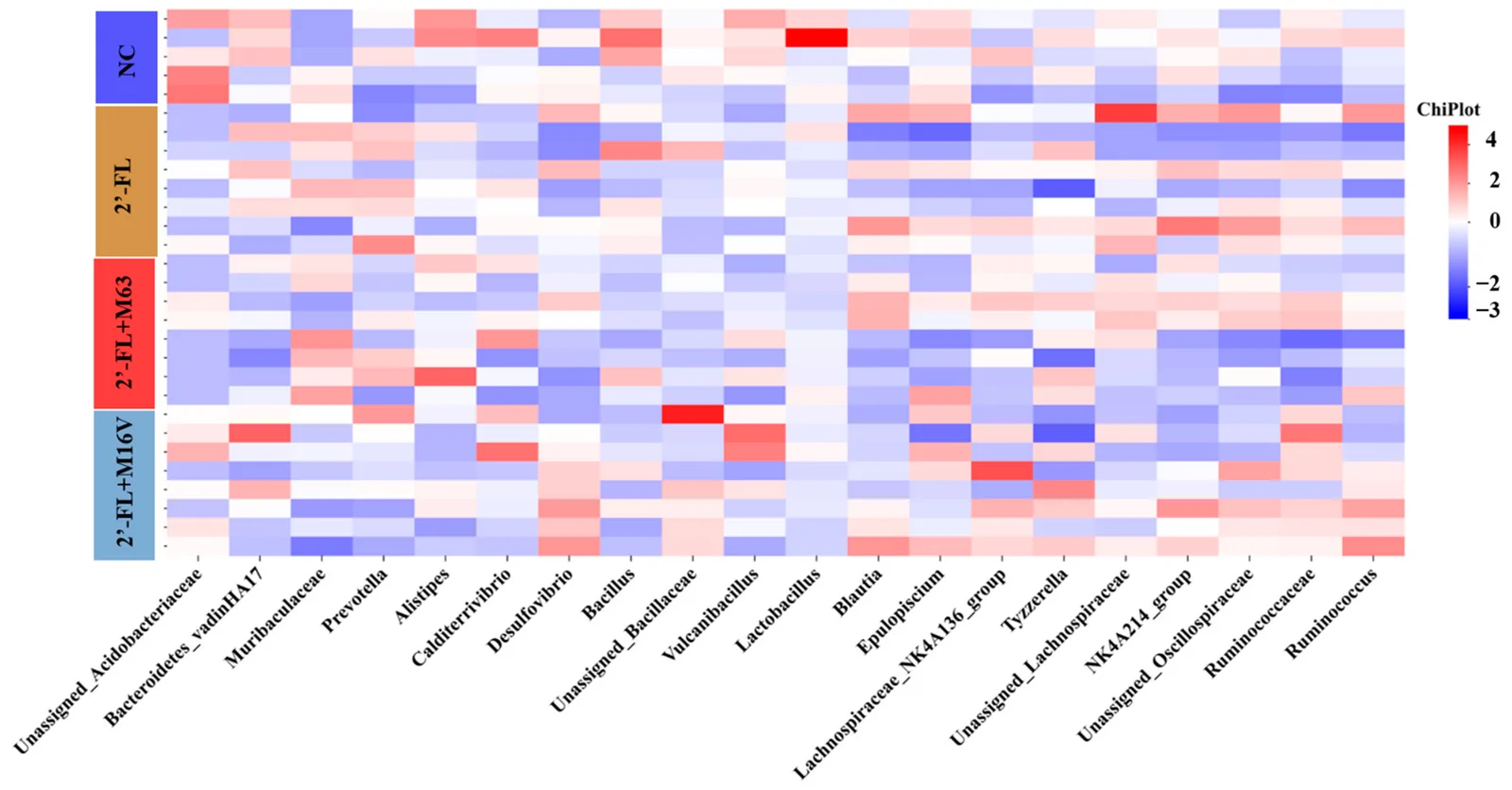
Composition of the gut microbiota in mice during the first week. *n* = 5–8. Negative control (NC), 2′-FL intervention group (2′-FL), 2′-FL + B. infantis M63 intervention group (2′-FL + M63), and 2′-FL + B. breve M16V intervention group (2′-FL + M16V).

In addition, fecal microbiota data from week 1 (prior to DSS modeling) indicated that oral administration of the tested substances had already exerted an initial modulatory effect on gut microbiota (Figure 8). Compared with the NC group, 2′-FL and synbiotic combinations altered the relative abundances of multiple genera, including *Muribaculaceae*, *Prevotella*, *Alistipes*, *Blautia*, *Lachnospiraceae_NK4A136_group*, and *Ruminococcaceae*. As this stage preceded DSS administration, microbiota changes primarily reflected the pre-modulatory effects of 2′-FL and synbiotics. Compared with week 2, separation of microbial communities among groups became more pronounced after DSS treatment, and the 2′-FL + M63 group further showed enrichment of *Muribaculaceae* and *Ruminococcaceae* and a reduction in Proteobacteria. These findings suggest that synbiotic combinations may have pre-shaped the gut microbiota prior to model induction and, following DSS exposure, promoted a microbial shift toward a state more conducive to oligosaccharide utilization and SCFA production.

### 3.7. Synbiotic Combinations Regulate Intestinal Lactate and Short-Chain Fatty Acid Metabolism

**Figure 9:**
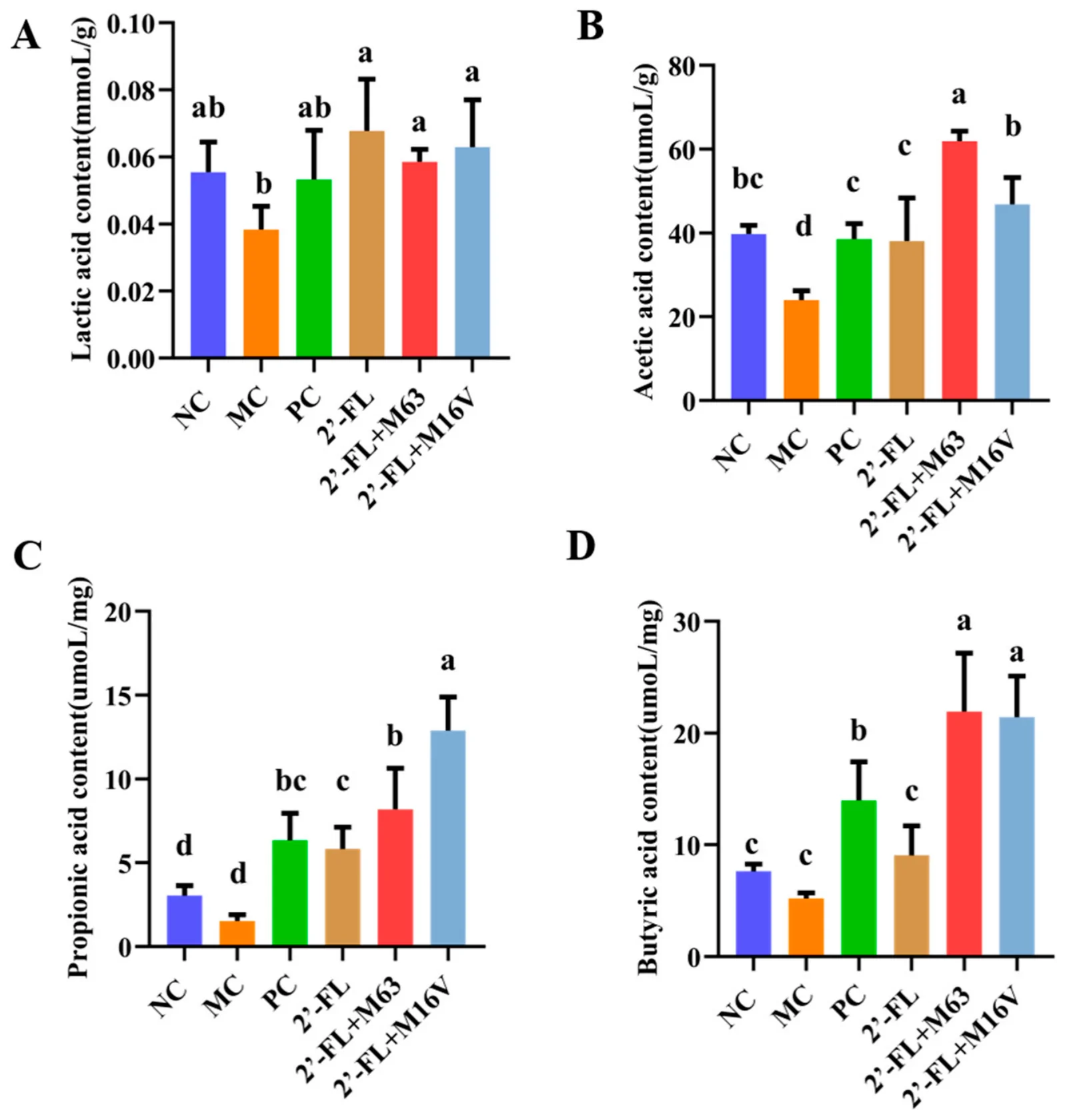
Synbiotics regulate the production of short-chain fatty acids in the gut. (**A**) Lactic acid. (**B**) Acetic acid. (**C**) Propionic acid. (**D**) Butyric acid. *n* = 4. Negative control (NC), model control (MC), positive control (PC), 2′-FL intervention group (2′-FL), 2′-FL + B. infantis M63 intervention group (2′-FL + M63), and 2′-FL + B. breve M16V intervention group (2′-FL + M16V). Note: Different letters (a–d) indicate statistically significant differences between groups (*p* < 0.05).

To further evaluate the effects of the synbiotic combinations on gut microbiota metabolic function, we measured lactic acid and major short-chain fatty acids in intestinal contents. As shown in Figure 9, compared with the NC group, the MC group exhibited significantly lower lactic acid and acetic acid levels (*p* < 0.05), indicating that DSS-induced colitis suppressed gut microbial fermentative activity. Compared with the MC group, the 2′-FL group showed significantly increased lactic acid, acetic acid, and propionic acid levels (*p* < 0.05). Meanwhile, the 2′-FL + M63 and 2′-FL + M16V groups exhibited significantly higher lactic acid, acetic acid, propionic acid, and butyric acid levels than the MC group (*p* < 0.05). In addition, acetic acid levels in the 2′-FL + M63 group were significantly higher than those in all other groups. In summary, 2′-FL and synbiotic interventions increased lactic acid and short-chain fatty acid (SCFA) levels in the gut. Furthermore, the 2′-FL + M63 group showed the strongest effect on increasing acetic acid levels.

## 4. Discussion

Colitis is a chronic intestinal disorder characterized primarily by persistent intestinal mucosal inflammation, epithelial barrier disruption, and gut microbiota dysbiosis. Its onset and progression are closely associated with host immune responses, intestinal epithelial integrity, and microbial metabolic function. The DSS-induced colitis model recapitulates key features of colitis, including epithelial injury and mucosal inflammation, and is widely used to evaluate potential anti-colitis bioactive agents [16]. In this study, DSS treatment led to body weight loss, an increased spleen index, and colon shortening in mice, accompanied by marked colonic tissue injury, crypt disruption, and reduced goblet cell abundance, indicating successful establishment of the colitis model. Both 2′-FL alone and its combinations with *B. infantis* M63 or *B. breve* M16V improved colitis-related phenotypes to varying degrees; among these treatments, the 2′-FL + M63 combination showed more pronounced effects in reducing the spleen index, restoring tissue architecture, and enhancing the expression of tight-junction- and mucus-barrier-related genes. Previous studies have shown that 2′-FL can alleviate DSS-induced intestinal inflammation by modulating the gut microbiota, promoting goblet cell recovery, and enhancing MUC2 secretion [8]. Furthermore, 2′-FL can ameliorate DSS-induced intestinal barrier damage and inhibit pro-inflammatory signaling pathways [9,10,11]. This study further demonstrates that the protective effects of 2′-FL against colitis, when combined with specific *Bifidobacterium* strains, are strain-dependent; in particular, the combination with *B. infantis* M63 may be more effective in alleviating DSS-induced intestinal inflammatory injury. The present findings should be interpreted not only as evidence of anti-colitic activity but also as preclinical proof-of-concept for a food-deliverable synbiotic ingredient system.

Persistent intestinal inflammation is a key driver of colitis progression. The pro-inflammatory cytokines TNF-α, IL-1β, and IL-6 promote immune-cell recruitment, epithelial injury, and amplification of the mucosal inflammatory cascade, whereas IL-10 is an important anti-inflammatory cytokine that maintains intestinal immune homeostasis [2]. In this study, DSS significantly increased IL-1β, IL-6, and TNF-α levels in colonic tissues and decreased IL-10 levels, indicating that DSS induced a typical intestinal pro-inflammatory response. 2′-FL, 2′-FL + M63, and 2′-FL + M16V all suppressed the increases in IL-6 and TNF-α levels, while the two synbiotic combinations also significantly reduced IL-1β levels, indicating that 2′-FL combined with *Bifidobacterium* exerted a broader inhibitory effect on pro-inflammatory cytokine production. Previous studies have reported that 2′-FL can suppress inflammatory responses associated with the TLR4/MyD88/NF-κB pathway and reduce colitis-related inflammation exacerbated by DSS or a high-fructose diet [8,11]. Furthermore, *B. breve* has been shown to alleviate colitis severity in DSS models and ameliorate inflammation and barrier injury [13,17]. Compared with 2′-FL alone, the synbiotic combinations in this study showed a more pronounced inhibitory effect on IL-1β, suggesting that *Bifidobacterium* may enhance the modulatory effect of 2′-FL on innate immune-mediated inflammatory responses.

Oxidative stress is a key pathological mechanism underlying inflammatory bowel disease. Excessive reactive oxygen species (ROS) can exacerbate lipid peroxidation, disrupt epithelial cell structure, and further amplify inflammation [18,19]. In this study, DSS significantly decreased catalase (CAT) and glutathione peroxidase (GSH-Px) activities while increasing malondialdehyde (MDA) levels, indicating marked oxidative stress in mouse colonic tissues. Both 2′-FL + M63 and 2′-FL + M16V significantly increased CAT and GSH-Px activities and reduced MDA levels, whereas 2′-FL alone did not significantly reduce MDA levels. These results suggest that *Bifidobacterium* strains may enhance the inhibitory effect of 2′-FL on lipid peroxidation. Previous studies have shown that natural nutritional components can protect intestinal tissues by enhancing antioxidant enzyme activities and regulating redox homeostasis [20]. Therefore, the alleviation of oxidative stress by the synbiotic combinations may be one of the key mechanisms underlying their improvement of DSS-induced colitis-related phenotypes and may act in concert with reduced pro-inflammatory cytokine production and restored gut-microbiota metabolism.

The intestinal epithelial barrier, which is maintained by tight junctions, the mucus layer, and the immune barrier, is a key structure that prevents luminal antigens and microorganisms from entering the lamina propria [21]. DSS can directly damage intestinal epithelial cells, leading to reduced tight-junction protein expression and increased intestinal permeability. In this study, DSS significantly reduced the mRNA expression of *Claudin-1*, *Claudin-2*, *Occludin*, and *ZO-1*, whereas 2′-FL + M63 significantly upregulated *Claudin-1*, *Claudin-2*, and *ZO-1*, and 2′-FL alone significantly increased *Occludin* expression. Tight-junction proteins include members of the claudin, *Occludin*, and zonula occludens (ZO) families. Among these proteins, *ZO-1* acts as a scaffold linking transmembrane tight-junction proteins to the cytoskeleton, whereas *Occludin* and claudins are involved in regulating paracellular permeability [3,22]. Previous studies have shown that 2′-FL can increase *ZO-1* and *Occludin* expression and alleviate DSS-induced colitis by restoring the intestinal mucosal barrier [23]. The results of this study are consistent with previous reports and further suggest that *Bifidobacterium* can enhance the regulatory effect of 2′-FL on the expression of tight-junction genes. Consistently, AB-PAS staining and mucus-barrier-related gene expression analysis showed that DSS reduced goblet cell abundance and mucus-positive signals, whereas 2′-FL + M63 significantly restored *TFF3* and *MUC2* expression. MUC2 is a major structural component of the colonic mucus layer, *TFF3* is involved in epithelial repair, and *RELMβ* is associated with mucosal defense and host–microbiota interactions [24,25]. Therefore, the protective effect of 2′-FL + M63 against colitis was reflected not only in the restoration of tight junctions but also in the enhancement of goblet cell secretory function and mucus layer integrity, which may reduce sustained epithelial injury caused by luminal irritants.

Gut microbiota dysbiosis is one of the key characteristics of colitis. Patients with IBD and animal models of colitis typically exhibit reduced microbial diversity, decreased abundance of short-chain fatty acid-producing bacteria, and a marked increase in Proteobacteria [26]. In this study, both 2′-FL and the two synbiotic interventions significantly increased the Shannon index, indicating improved microbial diversity. Phylum-level analysis showed that 2′-FL + M63 increased the relative abundance of *Bacteroidota* and decreased that of *Proteobacteria*. An increased abundance of *Proteobacteria* is considered a microbial marker of gut microbiota dysbiosis and epithelial dysfunction [27]. Therefore, its reduction suggests that 2′-FL + M63 may help restore gut-microbiota homeostasis. Genus-level and LEfSe analyses further revealed that 2′-FL + M63 enriched *Muribaculaceae* and *Ruminococcaceae*. *Muribaculaceae* is a common carbohydrate-utilizing bacterial family in the mouse gut and has the potential to degrade complex polysaccharides and produce metabolites such as propionic acid [28]. By contrast, *Ruminococcaceae* comprises various bacteria involved in fiber degradation and butyrate production and is closely associated with intestinal barrier function and anti-inflammatory effects [29,30]. Therefore, the microbial community shifts induced by 2′-FL + M63 may be more conducive to oligosaccharide metabolism and beneficial metabolite production, which is consistent with the increased acetic acid and butyrate levels observed in this study.

Short-chain fatty acids (SCFAs) are important metabolites that link the gut microbiota to host intestinal health. Acetic acid, propionic acid, and butyric acid not only serve as energy sources for colonic epithelial cells but also regulate inflammatory responses, enhance barrier function, and maintain immune homeostasis by activating G protein-coupled receptors and inhibiting histone deacetylases [31,32]. This study showed that DSS reduced lactic acid and SCFA levels in intestinal contents, whereas both 2′-FL and synbiotic interventions promoted the recovery of these metabolites. Notably, the 2′-FL + M63 combination significantly increased acetate levels, whereas the 2′-FL + M16V combination showed a more pronounced effect on propionate and butyrate levels. *Bifidobacterium* typically produces acetate and lactate by utilizing HMOs; these products can be cross-utilized by other gut microorganisms and further converted into metabolites such as butyrate [33,34]. *B. infantis* possesses a strong capacity to utilize HMOs, and its genome is enriched in genes involved in HMO transport and degradation [7,35]. Furthermore, synbiotics composed of HMOs and *B. infantis* have been shown to promote the colonization of specific bacterial communities and reshape metabolic functions in the adult gut [36,37]. Therefore, the increase in acetate induced by 2′-FL + M63 in this study may be attributable to the efficient utilization of 2′-FL by M63 and its cross-feeding interactions with bacterial taxa such as *Muribaculaceae* and *Ruminococcaceae*.

This study has several limitations. The gut microbiota data were analyzed using operational taxonomic unit (OTU) clustering at 97%. Although this approach remains commonly used, amplicon sequence variant (ASV)-based methods generally provide higher sequence resolution and improved reproducibility. OTU clustering may merge closely related sequences, obscure low-abundance variants, and consequently underestimate subtle differences in microbial community structure. Therefore, the taxonomic findings should be interpreted with appropriate caution. Future studies should adopt ASV-based pipelines, such as DADA2 or Deblur, and incorporate metagenomic and metabolomic analyses to improve taxonomic resolution and functional interpretation.

## 5. Conclusions

Overall, this study demonstrates that *Bifidobacterium* can alleviate DSS-induced colitis in mice by facilitating various functional mechanisms associated with 2′-FL, including suppression of excessive pro-inflammatory cytokine production, alleviation of oxidative stress, restoration of tight junctions and the mucus barrier, reshaping of gut microbiota composition, and promotion of lactic acid and short-chain fatty acid (SCFA) production. Among the tested interventions, the 2′-FL + M63 combination showed the greatest efficacy in improving the spleen index, mucus-barrier integrity, tight-junction gene expression, gut microbiota composition, and acetate production. In conclusion, these findings provide experimental evidence for developing functional synbiotic foods based on 2′-FL and specific *Bifidobacterium* strains.

## Figures and Tables

**Table 1:** Experimental grouping and treatment.

Grouping	Adaptation Period	1 Week	2 Weeks
NC	Free food and water	Free drinking water + 0.2 mL lactose solution via gavage	Free drinking water + 0.2 mL lactose solution via gavage
MC	Free food and water	Free drinking water + 0.2 mL lactose solution via gavage	Administer 2.5% DSS via drinking water + 0.2 mL lactose solution via gavage
PC	Free food and water	Free drinking water + 0.2 mL lactose solution via gavage	2.5% DSS in drinking water + 0.2 mL 5-aminosalicylic acid via gavage
2′-FL	Free food and water	Free drinking water + 0.2 mL 2′-FL via gavage	2.5% DSS in drinking water + 0.2 mL 2′-FL via gavage
2′-FL + M63	Free food and water	Free drinking water + 0.2 mL 2′-FL + M63 via gavage	2.5% DSS in drinking water + 0.2 mL 2′-FL + M63 via gavage
2′-FL + M16V	Free food and water	Free drinking water + 0.2 mL 2′-FL + M16V via gavage	2.5% DSS in drinking water + 0.2 mL 2′-FL + M16V via gavage

**Table 2:** Primer information.

Target Gene	Primer Sequence (5′-3′)	Reverse Sequence (5′-3′)
*Claudin-1*	TTCGCAAAGCACCGGGCAGATACA	GCCACTAATGTCGCCAGACCTGAAA
*Claudin-2*	TGCGACACACAGCACAGGCATCACC	TCAGGAACCAGCGGCGAGTAGAA
*Occludin*	AACAACCCCTTCCAAGTTCC	CTCCCAGAGTTCCGATTCAC
*ZO-1*	GGGAGGGTCAAATGAAGACA	GGCATTCCTGCTGGTTACAT
*MUC 2*	GCTGACGAGTGGTTGGTGAATG	GATGAGGTGGCAGACAGGAGAC
*TFF3*	CCCTGGTGCTTCAAACCTCT	GGGATGCTTGCTACCCTTG
*RELMβ*	CTGATAGTCCCAGGGAACGC	GTCTGCCAGAAGACGTGACA
*GAPDH*	AAGCCCATCACCATCTTCCA	CACCAGTAGACTCCACGACA

## Data Availability

The original contributions presented in this study are included in the article. Further inquiries can be directed to the corresponding authors.
